# The hidden pathways to internalized weight bias: from insecure attachment to depressive self-schemas

**DOI:** 10.3389/fpsyt.2025.1703650

**Published:** 2025-11-10

**Authors:** Emanuela Bianciardi, Rossella Mattea Quinto, Ester Longo, Federica De Angelis, Alberto Siracusano, Cinzia Niolu, Giorgio Di Lorenzo

**Affiliations:** 1 Chair of Psychiatry, Department of Systems Medicine, University of Rome Tor Vergata, Rome, Italy; 2 Psychiatric and Psychological Unit, Fondazione Policlinico Tor Vergata, Rome, Italy; 3 Department of Health and Life Sciences, European University of Rome, Rome, Italy; 4 IRCCS Fondazione Santa Lucia, Rome, Italy

**Keywords:** internalized weight bias, body image dissatisfaction, depression, attachment style, eating disorder, self-stigma, overweight and obesity

## Abstract

**Background:**

Internalized weight bias (IWB) is associated with adverse physical and psychopathological outcomes, yet the cognitive and emotional mechanisms underlying its development in non-clinical populations remain insufficiently understood. This study examined whether attachment insecurity, depressive symptoms, and alexithymia were related to IWB in adults with overweight/obesity, and tested a parallel mediation model of depressive symptoms and alexithymia in the link between attachment insecurity and IWB.

**Methods:**

194 Italian adults (75% female; Mage = 37.6, SD = 14.7; BMI ≥ 25) completed an online survey including self-report measures of IWB, attachment style, depression, alexithymia, eating disorder risk, and body dissatisfaction. Hierarchical regression models were conducted to identify predictors of IWB, followed by mediation analyses (PROCESS Model 4) to test indirect effects.

**Results:**

The final regression model explained 74% of the variance in IWB. Significant predictors included body dissatisfaction (*β* = .43, *p* <.001), cognitive depressive symptoms (*β* = .22, *p* <.001), and anxious attachment (*β* = .30, *p* <.001). Difficulty describing feelings was unexpectedly inversely associated with IWB (*β* = –.11, *p* = .04). Mediation analyses revealed that cognitive depressive symptoms partially mediated the relationship between anxious attachment and IWB, whereas alexithymia dimensions did not.

**Conclusion:**

Findings highlighted cognitive depressive symptoms as a central pathway linking insecure attachment to IWB, while suggesting a paradoxical protective role for alexithymic difficulties in emotional expression. We emphasized the importance of considering individual vulnerability factors—particularly relational insecurity and depressive cognitions—in theoretical models of IWB and in the design of targeted clinical interventions.

## Introduction

Weight stigma is a pervasive and widespread public health problem with harmful consequences for individuals’ well-being, affecting predominantly — but not exclusively — the person with overweight and obesity (1). It occurs when a person’s worth, abilities, and personal characteristics are rated or assumed based solely on their body weight (2). Weight stigma may begin in early childhood, within the family, at school, among peers, in the workplace, and in healthcare settings (3, 4). It is expressed in a wide spectrum of forms, such as comments, unsolicited advice, and judgments, to avoidance of eye contact, gossip, social exclusion and isolation, through to harassment and bullying (5, 6). The negative consequences for the individual are incalculable; some are striking, as suicide (7), while others are more insidious yet corrosive, shaping the personality over time, leading to the internalization of weight bias (8). The victim directs negative weight-based stereotypes toward themself, until they sabotage confident behavior in social situations (9). The prevalence of internalized weight bias (IWB) is globally underestimated and varies across clinical and non-clinical populations, age groups, with higher rates observed among females and gender minorities (10). A US study in a large non-clinical adult population found a prevalence of about 24%, regardless of body mass index (BMI) (11), while another reported that high levels of IWB are present in between one-fifth and one-half of adults across different body weight categories (12).

IWB has been associated with feeding and eating disorders (FEDs), including purging-type anorexia nervosa (13), bulimia nervosa (14), binge eating disorder (BES) (15), and food addiction (FA) (16), with variations in the strength and direction of these associations across conditions (17). A recent review of up to 200 studies found a consistent relationship between greater weight stigma and more disordered eating cognitions and behaviors (18). However, the mechanisms through which cognitive and emotional factors contribute to IWB remain less explored, particularly in non-clinical populations.

The development of internalized weight stigma is conceived as the process of directing and internalizing one’s own experiences of weight-related stigma (19). Nevertheless, research has shown that even seemingly non-personal forms of stigma, such as negative weight-related messages within one’s social environment, can be equally harmful, as they promote self-blame and increase the likelihood of internalization (20). This highlights the potential role of shared vulnerability factors, which constitute the focus of this investigation and may lay the groundwork for the internalization of weight bias.

According to attachment theory, early relationships with primary caregivers are fundamental in shaping enduring patterns of beliefs and behaviors in adult close relationships and in response to stress (21). Insecure attachment styles are associated with increased risk of a broad range of mental and physical health problems, as these patterns may impair coping strategies, interpersonal expectations, self-concept, and emotional regulation (22, 23). Prior evidence indicates that insecure attachment heightens sensitivity to external evaluation and undermines self-esteem and adaptive coping (24). Therefore, individuals with insecure attachment style may be more sensitive and vulnerable to negative weight-related messages.

Moreover, depressive symptoms and deficits in emotion regulation may facilitate the development of IWB. On the one hand, depression can promote the assimilation of negative weight-based stereotypes toward the self, due to the diminished self-esteem, a pessimistic perspective on the future, and the perception of one’s weight as an unchangeable and uncontrollable condition (25).

On the other hand, emotion dysregulation stemming from impairments in the ability to regulate emotions in an adaptive manner (26) has been linked to IWB, particularly among individuals with FEDs (27).

Alongside deficits in emotion regulation, alexithymia—characterized by difficulties in identifying and describing emotions, a limited capacity for emotional awareness, and a tendency toward externally oriented thinking (28) may also contribute to IWB. By limiting emotional awareness, alexithymia can foster maladaptive coping strategies and ultimately increase susceptibility to adopting negative weight-based stereotypes as self-defining. Accordingly, non-judgmental awareness and acceptance of one’s feelings were associated with lower IWB, both with and without co-occurring FEDs (29).

Finally, depressive symptoms, alexithymia, and attachment insecurity have all been connected with negative self-schema and body image concerns, suggesting their potential role in IWB (30–32).

Indeed, body image dissatisfaction (BID) involving negative emotional, cognitive, and behavioral facets of body size and shape (33) is conceptually close to IWB (34), as both entail the application of undesirable body-related stereotypes to the self (35).

### Aim and hypotheses

This study aimed to examine the cognitive and emotional correlates of IWB in a non-clinical adult sample with overweight or obesity. Based on prior literature, we hypothesized that insecure attachment style, higher levels of depressive symptoms, and alexithymia would be associated with higher IWB scores, after controlling for age, sex, risk of FEDs, and BID. Further, we aimed to test a mediation model examining whether cognitive depressive symptoms and alexithymia mediated the association between attachment insecurity and IWB.

## Materials and methods

### Participants

The data for the present study were derived from a larger investigation on FEDs in the general population, which was conducted between September and December 2024, administering an anonymous online survey using the free software Google Forms^®^ (Google LLC, Mountain View, CA, USA). Participants were recruited using a convenience and snowball sampling strategy. The survey link was shared on university students’ social networks and complemented by poster advertisements placed in supermarkets in Rome, Italy. Online consent was obtained from the participants before data collection; they were allowed to terminate the survey at any time. Only one submission per participant was allowed, verified through quality control procedures. The study received approval from the local Institutional Review Board and was conducted in accordance with the ethical standards of the 1964 Declaration of Helsinki.

Inclusion criteria included: (1) to provide informed consent before survey participation; (2) to reside, study, or work in Italy; (3) to have sufficient proficiency in Italian to independently complete the questionnaire; and (4) to complete all required measures, including the sociodemographic form. Exclusion criteria included the absence of informed consent, incomplete survey responses, missing data on key variables, and duplicate or repeated submissions identified through quality checks.

For the current analysis, only individuals with a BMI equal to or greater than 25 were selected; therefore, the final sample included 194 participants (47 males, 146 females, one not specified; *M*_age_ = 37.61, *SD* = 14.7).

### Measures

Participants were asked to complete a checklist assessing sociodemographic variables (i.e., gender, age, marital status, years of education, occupation, and medication). Self-reported height and weight were used to calculate the BMI. The following psychometrics were administered:

The Italian Weight Bias Internalization Scale (36) assessed how individuals with overweight or obesity internalize negative weight-based stereotypes. The scale, originally composed of 11 items (37), was reduced to 9 items by removing items 1 and 9. This shorter version demonstrated good internal reliability and convergent validity in people with overweight and obesity (36). In our sample, Cronbach’s alpha was 0.95 for the total score.

The Eating Attitudes Test – 26 (38, 39) measured disordered eating symptoms; scores ≥20 indicated risk for an eating disorder, with good internal consistency (α = .86).

The Body Shape Questionnaire (40, 41) is a 34-item tool assessing body dissatisfaction (α = .98).

The Toronto Alexithymia Scale (42, 43) is a 20-item questionnaire assessing alexithymia across three dimensions: Difficulty Identifying Feelings (DIF), representing problems in recognizing emotions and distinguishing them from bodily sensations; Difficulty Describing Feelings (DDF), that refers to the challenges in verbally expressing emotions, and Externally Oriented Thinking (EOT), describing the tendency to focus on external, practical details rather than inner emotions. The internal consistency was acceptable (DIF: α = 0.86; DDF: α = 0.78; EOT: α = 0.60).

The Beck Depression Inventory – II (44, 45) is a 21-item instrument assessing cognitive-affective (e.g., sadness, pessimism, self-criticism) and somatic-performance (e.g., fatigue, changes in sleep and appetite, work difficulties) symptoms of depression (α = .89; α = .60).

The Attachment Style Questionnaire (46, 47) is a 26-item measure assessing adult attachment in relational contexts. In this study, we adopted the bi-dimensional structure proposed by Fossati et al. (47), which conceptualizes attachment as two higher-order factors: avoidant (α = 0.84) and anxious attachment (α = 0.90).

### Statistical analyses

All analyses were performed with SPSS for Windows 26.0. All data were checked for normality, and no variables reported skewness and kurtosis values higher than 1. Thus, parametric analyses were carried out. Categorical variables were presented as counts and percentages, while continuous variables were described using means and standard deviations.

We performed a hierarchical multiple regression analysis to investigate potential factors influencing weight bias. Multivariate outliers were detected using Mahalanobis Distance (*D²*), applying the criterion where cases with a *D²* value exceeding the threshold of 26.13 (i.e., *D²* value at *p* < 0.05, with 8 degrees of freedom) (48) were considered outliers. The sequence of independent variables in the hierarchical regressions was determined based on the study’s objectives and theoretical rationale. The first step involved covariates, including sex, age, and BSQ dichotomized (0= no or mild concern with shape; 1= moderate-to-marked concern with shape). In the second step, variables indicating the presence or risk of eating disorders were entered as dichotomous predictors (0 = below cut-off; 1 = above cut-off). Specifically, the EAT-26 (cut-off ≥ 20) was used to identify individuals at risk of eating disorders. The third step included alexithymia, as measured by TAS-20, while in the fourth step, depressive symptoms, both cognitive and somatic dimensions, were entered in the model. Finally, in the fifth step, the anxious and avoidant attachment styles, assessed through the ASQ, were included in the model.

Sensitivity analyses using continuous BSQ and EAT-26 scores yielded comparable results (data not shown), supporting the robustness of our findings. In the main analyses, these measures were dichotomized to identify moderate-to-severe body-image dissatisfaction and eating-disorder risk, respectively.

To examine the hypothesized relationships among the variables, we employed Model #4 of the PROCESS macro for SPSS (49), which tests a parallel mediation model. The attachment style was entered as the focal predictor, while weight bias was entered as the outcome variable. Emotional-affective variables (e.g., depression, alexithymia) were included as parallel mediators. Only those variables that were statistically significant in preliminary regression analyses were included in the final model. Indirect effects were estimated using bootstrapping with 5,000 resamples, generating 95% bias-corrected confidence intervals. Effects were considered statistically significant when the confidence intervals did not include zero.

## Results

### Demographic and clinical characteristics

Of the 194 participants with overweight/obesity, about 75% were females (*N* = 146), with a mean age of 37 years. Most participants were single (*N* = 92; 47.4%) and employed at the time of the study (*N* = 113; 60.1%). Approximately 45% held a high school diploma (*N* = 89), while about 48% had completed a university degree (*N* = 94). Most participants regularly took no medication (*N* = 101; 52.3%; for further details, see **Table 1**).

**Table 1:** Sociodemographic characteristics of the sample.

Variable, N (%)	Total sample
	(*N* = 194)
Age, *M* (*SD*)	37.61 (14.7)
Sex	
Male	47 (24.2)
Female	146 (75.3)
Not-specified	1 (0.5)
Education	
Middle school diploma	11 (5.7)
High school diploma	89 (45.9)
Graduate/post-graduate degree	94 (48.4)
Marital status	
Single	92 (47.4)
Married/cohabitant	91 (46.9)
Divorced/separated	11 (5.7)
Occupation	
Not employed	75 (39.9)
Employed	113 (60.1)
Regular medication use	
Yes	92 (47.7)
No	101 (52.3)

### Psychopathological factors associated with weight-bias internalization

**Table 2** presents the results of the hierarchical regression analysis exploring the associations between IWB and a range of psychopathological and clinical predictors, while controlling for age, gender, and body dissatisfaction, as measured by BSQ. In the first step, moderate-to-marked concern with body explained a substantial portion of the variance in weight bias scores, with the model accounting for 58% of the variance (*R²* = .58; *p* <.001). In the second step, the inclusion of eating disorder risk (EAT-26) led to a small but significant increase in explained variance (Δ*R²* = .02), with EAT-26 emerging as a significant predictor (*β* = .18; 95%CI 3.51, 13.51; *p* <.001). In the third step, the three alexithymia dimensions were added, but their contribution to the model was minimal (*ΔR²* = .01) and not statistically significant. None of the alexithymia subscales reached significance at this stage. In the fourth step, the two dimensions of BDI-II explained an additional 8% variance in weight bias scores (Δ*R²* = .08, *p* <.001). The cognitive subscale of the BDI-II emerged as a strong and significant predictor of IWB (*β* = .34; 95%CI 0.52, 1.13; *p* <.001), while the somatic subscale was not significant. In the final step, attachment styles were included, resulting in an additional 5% of explained variance (Δ*R²* = .05, *p* <.001). Among the attachment dimensions, only anxious attachment significantly predicted IWB (*β* = .30; 95%CI 0.16, 0.38; *p* <.001), while avoidant attachment did not (*β* = .07; *n.s.*).

**Table 2:** Regression analysis for WBIS.

		*B*	95.0% CI		*SE B*	*β*	*R^2^*	Δ*R^2^*
			*LI*	*LS*				
Step 1							.58	.58^***^
	(Constant)	18.74	10.93	26.54	3.95			
	Age	-0.07	-0.18	0.05	0.06	-.06		
	Gender	2.77	-0.98	6.53	1.90	.07		
	BSQ	23.75	20.35	27.15	1.72	.73^***^		
Step 2							.60	.02^***^
	(Constant)	18.10	10.51	25.69	3.84			
	Age	-0.06	-0.17	0.06	0.06	-.05		
	Gender	2.84	-0.81	6.48	1.85	.07		
	BSQ	21.06	17.40	24.72	1.85	.65^***^		
	EAT-26	8.51	3.51	13.51	2.53	.18^***^		
Step 3							.61	.01
	(Constant)	14.11	4.00	24.23	5.13			
	Age	-0.05	-0.17	0.06	0.06	-.05		
	Gender	2.59	-1.08	6.26	1.86	.07		
	BSQ	19.91	16.03	23.80	1.97	.61^***^		
	EAT-26	7.95	2.79	13.11	2.61	.17^***^		
	TAS_DIF	0.25	-0.04	0.55	0.15	.11		
	TAS_DDF	0.05	-0.44	0.34	0.20	.01		
	TAS_EOT	0.06	-0.32	0.43	0.19	.02		
Step 4							.69	.08^***^
	(Constant)	14.07	5.00	23.15	4.60			
	Age	-0.03	-0.13	0.08	0.05	-.02		
	Gender	1.78	-1.52	5.09	1.67	.05		
	BSQ	16.29	12.62	19.96	1.86	.50^***^		
	EAT-26	3.19	-1.67	8.05	2.46	.07		
	TAS-20 DIF	0.05	-0.23	0.32	0.14	.02		
	TAS-20 DDF	0.08	-0.43	0.27	0.18	-.02		
	TAS-20 EOT	0.02	-0.32	0.35	0.17	.00		
	BDI-II Cognitive	0.83	0.52	1.13	0.15	.34^***^		
	BDI-II Somatic	0.27	-0.32	0.87	0.30	.05		
Step 5							.74	.05^***^
	(Constant)	-5.84	-16.95	5.26	5.62			
	Age	0.02	-0.08	0.12	0.05	.02		
	Gender	1.35	-1.69	4.40	1.54	.04		
	BSQ	13.83	10.37	17.30	1.75	.43^***^		
	EAT-26	4.08	-0.37	8.53	2.25	.09		
	TAS-20 DIF	0.05	-0.30	0.20	0.13	-.02		
	TAS-20 DDF	0.35	-0.68	-0.01	0.17	-.11^*^		
	TAS-20 EOT	0.08	-0.22	0.39	0.16	.02		
	BDI-II Cognitive	0.53	0.23	0.83	0.15	.22^***^		
	BDI-II Somatic	0.21	-0.34	0.75	0.28	.04		
	ASQ Avoidant Attachment	0.11	-0.05	0.26	0.08	.07		
	ASQ Anxious Attachment	0.27	0.16	0.38	0.06	.30^***^		

*p < 0.05; ** *p* < 0.01; *** *p* < 0.001; BSQ = Body Shape Questionnaire; EAT-26 = Eating Attitudes Test – 26; TAS = Toronto Alexithymia Scale; BDI-II = Beck Depression Inventory – II; ASQ = Attachment Styles.

The model explained 74% of the variance of IWB when all predictors were included. Significant predictors in the final model included body dissatisfaction (BSQ; *β* = .43; 95%CI 10.37, 17.30; *p* <.001), cognitive depressive symptoms (BDI-II Cognitive; *β* = .22; 95%CI 0.23, 0.83; *p* <.001), anxious attachment (ASQ; *β* = .30; 95%CI 0.16, 0.38; *p* <.001), and difficulty describing feelings (TAS-20 DDF; *β* = -.11; 95%CI -0.68, -0.01; *p* = .04). The results indicated that greater body shape dissatisfaction, cognitive depressive symptoms, and anxious attachment significantly predicted higher levels of weight bias internalization. Interestingly, lower difficulties describing feelings were also associated with higher weight bias.

### Parallel mediation model

**Figure 1:**
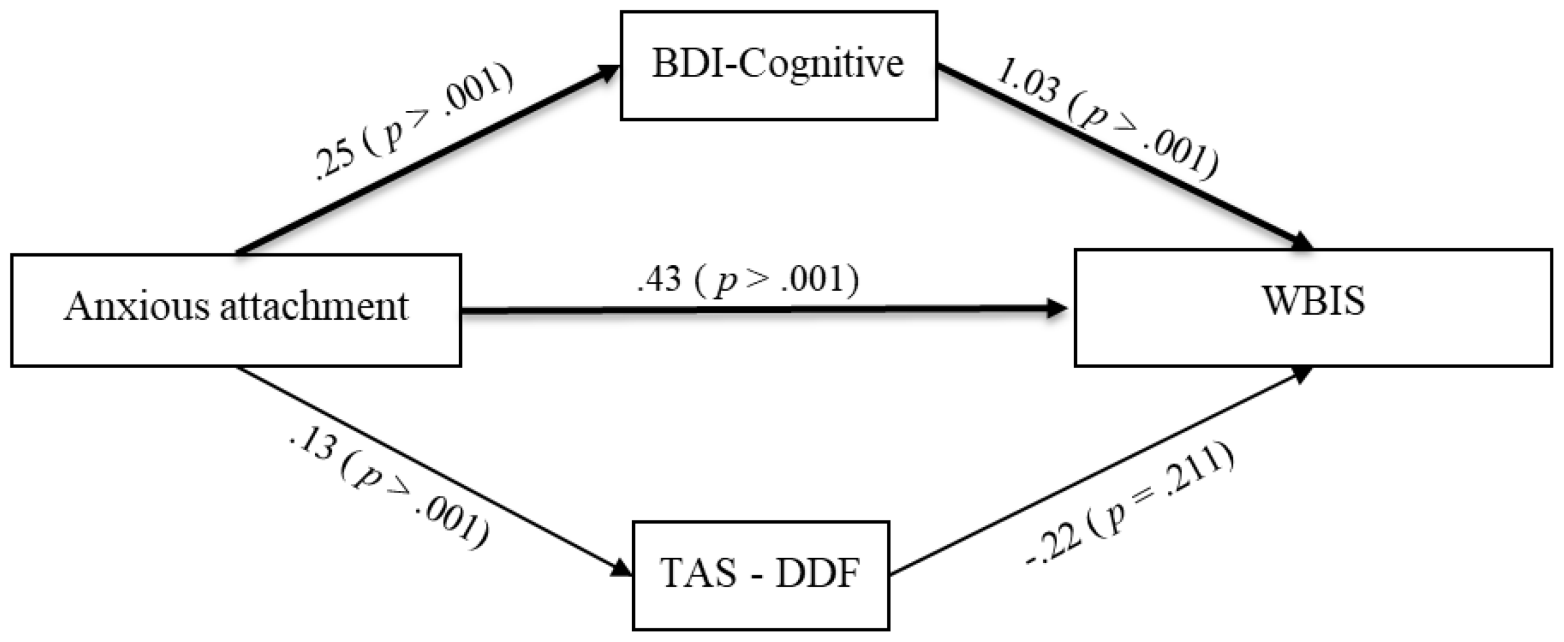
Parallel mediation model (PROCESS Model #4) testing the effect of anxious attachment on weight bias internalization via cognitive depressive symptoms (BDI-Cognitive subscale) and difficulty in describing feelings (TAS-20-DDF subscale). Standardized coefficients (*β*) and *p*-values are reported. Bold lines indicate significant mediation paths. Sample size: *N* = 183.

A parallel mediation analysis was conducted using PROCESS Model 4 (49) on a sample of 183 patients to examine whether cognitive depressive symptoms (BDI-II Cognitive subscale) and difficulty describing feelings (TAS-20 DDF subscale) mediated the relationship between anxious attachment and internalized weight bias (WBIS Total score; see **Figure 1**).

Results showed that anxious attachment was significantly associated with both mediators: higher anxious attachment predicted higher cognitive depressive symptoms (*B* = 0.254, *SE* = 0.022, *p* <.001) and greater difficulty describing feelings (*B* = 0.126, *SE* = 0.018, *p* <.001). Significant direct effect emerged between cognitive depressive symptoms and IWB scores (*B* = 1.029, *SE* = 0.142, *p* <.001); however, the direct effect of difficulty describing feelings on IWB was not statistically significant (*B* = –0.215, *SE* = 0.171, *p* = .211).

The total effect of anxious attachment on IWB was significant (*B* = 0.664, *SE* = 0.047, p <.001), and the direct effect remained significant after controlling for the mediators (*B* = 0.430, *SE* = 0.059, *p* <.001), indicating partial mediation. The full model predicting WBIS from Anxious Attachment, BDI Cognitive dimension, and TAS-DDF was significant, *F*(3, 179) = 101.31, p <.001, and explained 62.93% of the variance in WBIS total score (*R²* = .6293). Bootstrap analysis (5,000 samples) revealed a significant total indirect effect of anxious attachment on weight bias through the mediators (Effect = 0.234, BootSE = 0.048, 95% CI[0.140, 0.328]). Specifically, the indirect effect through cognitive depressive symptoms was significant (Effect = 0.261, BootSE = 0.040, 95% CI[0.186, 0.342]), while the indirect effect through difficulty describing feelings was not (Effect = –0.027, BootSE = 0.022, 95% CI[–0.071, 0.015]).

These results suggest that cognitive symptoms of depression partially mediated the relationship between anxious attachment and IWB. In contrast, difficulty describing feelings did not play a significant mediating role.

## Discussion

This study investigated the cognitive and emotional correlates of IWB in a non-clinical adult sample with overweight and obesity. Our findings supported the hypothesis that only cognitive depressive symptoms, rather than somatic ones, along with attachment insecurity, were positively associated with IWB, independently of body image dissatisfaction and feeding and eating disorder risk. Notably, greater difficulty in identifying feelings—an alexithymic feature reflecting impaired emotional mentalization—was inversely associated with IWB. Furthermore, our psychopathological model showed that attachment insecurity was linked to IWB both directly and indirectly through the effect of depressive symptoms. Contrary to expectations, alexithymia did not mediate this relationship.

This study adds novelty by examining attachment style as a possible basic dimension of personal vulnerability. Attachment style plays a central role in interpersonal relationships, shaping individuals’ expectations about themselves and others (50); for this reason, it is important in the field of IWB. While prior research has reported associations between depression and IWB (51), our study offers an original contribution by showing that depression functions as the cognitive link between relational insecurity and IWB.

Individuals with insecure attachment styles may exhibit heightened anxiety and vigilance toward external judgment due to poor self-esteem and a strong need for others’ approval (52), thereby increasing the expectation of rejection based on body size. Depressive cognitions such as self-blame, hopelessness, and beliefs about the immutability of one’s situation can further affect feelings of powerlessness, sustained by repetitive and self-perpetuating negative thoughts that are typical of depression. Unexpectedly, the difficulty describing feelings dimension of alexithymia—conceptually linked to interpersonal and social functioning—appears to play a distinctive role in the self-stigma process (53). One possible explanation is that reduced emotional awareness may buffer sensitivity to external evaluation and thereby attenuate the impact of stigmatizing experiences. However, this interpretation should be considered tentative, as empirical evidence on this specific association remains scarce. We highlight that our results stimulate further theoretical reflection on IWB, emphasizing the role of individual vulnerability. Moreover, they carry clinical implications, since depression is a well-known and modifiable factor (54).

Whereas many studies have primarily focused on external risk factors, with large-scale longitudinal studies identifying sources of weight stigma in peers, schools, families, and workplaces—as well as precipitating experiences such as weight-related bullying (55)—comparatively less attention has been devoted to how individuals navigate and internalize stigma from their own psychological perspective. Considering the individual’s history and cognitive appraisals when examining how weight stigma becomes internalized challenges the linear conceptualization of self-stigma. The most widely adopted framework has been Corrigan et al.’s model of self-stigma (56), which describes progressive stages of stereotype awareness, personal agreement, self-concurrence, and subsequent impairment of well-being. By contrast, our findings support evidence showing that not all individuals who experience weight stigma will necessarily internalize it, with reports showing that between 21.8% and 41.7% of individuals with above-average levels of weight bias internalization did not account for prior experiences of weight stigma, including teasing from peers or family, weight-based discrimination, or unfair treatment due to their body weight (12). Moreover, it has been elucidated that individuals may not completely agree with negative stereotypes (19), and IWB can also occur among those who do not endorse or apply negative weight-based stereotypes to themselves (57), suggesting a complex interplay between vulnerability factors, self-defense mechanisms, and internalization processes. Thus, IWB should not be conceptualized as a purely passive phenomenon. From a clinical standpoint, our work supports the literature underscoring that beyond the experience of weight stigma, its internalization is harmful for physical and mental health and needs to be carefully considered.

There is consistent evidence linking IWB to poorer physical health, such as cardiometabolic health, somatic symptoms, anthropometric indices of metabolic risk, and weight cycling (i.e., less weight maintenance from pre- to post-weight loss intervention (58). Furthermore, multiple adverse mental health outcomes have been consistently observed in people with IWB, including disinhibited eating behaviors, anxiety, social functioning, and quality of life (59). These associations appear to be stronger among individuals under 18 (60), a developmental stage in which personal identity is still being formed (61). In older individuals, WBIS scores appeared to converge with the dimension of self-esteem, likely reflecting the cumulative effect of repeated experiences (62).

From a clinical perspective, the repetitive negative cognitions may also reduce help-seeking behaviors and contribute to clinical attrition, with individuals believing that “nothing can be done” (63, 64). This is consistent with recent findings that IWB is significantly associated with attrition in weight-loss treatments (65).

## Limitations and future directions

This study has several limitations that should be acknowledged. First, the cross-sectional design precludes causal inference about the observed associations, including the hypothesized mediation pathway. Second, we included only participants with overweight or obesity, since the Italian version of the IWB measure has not been validated for individuals with a BMI < 25. Nonetheless, substantial evidence indicates that IWB occurs across all weight categories, which we were therefore unable to examine. Third, all measures were based on self-report instruments, which may be influenced by reporting bias. The predominance of female participants (75%) limits generalizability, as sex/gender differences may influence experiences of weight stigma. Moreover, sexual orientation and socioeconomic status were not assessed, despite the established vulnerability of gender minorities to higher levels of internalized weight stigma (66). This limitation highlights the need for future studies to include gender minorities. Finally, recruitment via convenience and snowball sampling (university social networks and supermarkets) may have introduced self-selection bias and limited sample representativeness.

Given the possible severe outcomes of IWB—including impaired mental health, disordered eating, and even suicidality—future research should adopt longitudinal designs to clarify causal pathways and examine how these cognitive and emotional correlates influence health-related outcomes over time, particularly among vulnerable youth, and by stratifying participants into age and BMI groups.

Furthermore, studies should focus on risk factors and investigate psychological processes that may facilitate resistance to internalized weight stigma, thereby informing the development of preventive and therapeutic interventions.

## Conclusions

In summary, this study provided novel evidence on the role of individual vulnerabilities in IWB among non-clinical adults with overweight and obesity. Anxious attachment emerged as a key relational factor that directly and indirectly increased IWB through cognitive depressive symptoms, underscoring the role of maladaptive self-schemas in sustaining weight-related self-stigma. Unexpectedly, difficulties in describing emotions appeared inversely related to IWB, suggesting that limited emotional awareness may sometimes buffer against the internalization of negative stereotypes.

Clinically, these findings supported the need to target depressive cognitions and attachment-related insecurities within therapeutic interventions addressing weight stigma. Theoretically, they called for a more comprehensive, non-linear conceptualization of IWB that integrates external stigmatizing influences with internal vulnerability factors. Future longitudinal research is warranted to clarify causal pathways and to explore resilience processes that may protect against self-stigma, thereby informing prevention and treatment strategies.

## Data Availability

The raw data supporting the conclusions of this article will be made available by the authors, without undue reservation.
